# Effect of Plant-Based Mouthwash (*Morinda citrifolia* and *Ocimum sanctum*) on TNF-α, IL-α, IL-β, IL-2, and IL-6 in Gingival Crevicular Fluid and Plaque Scores of Patients Undergoing Fixed Orthodontic Treatment

**DOI:** 10.3390/medicina59111968

**Published:** 2023-11-08

**Authors:** Muhammad Abdullah Kamran, Abdullah A. Alnazeh, Salem Almoammar, Mohammad Almagbol, Eisha Abrar Baig, Mohammad Raji Alrwuili, Mohammed Ahmed Aljabab, Ibrahim Alshahrani

**Affiliations:** 1 Department of Pedodontics and Orthodontic Sciences, College of Dentistry, King Khalid University, Abha 62529, Saudi Arabia; aalnazh@kku.edu.sa (A.A.A.); smalmoamr@kku.edu.sa (S.A.); ishahrani@kku.edu.sa (I.A.); 2 Department of Community and Periodontics, Faculty of Dentistry, King Khalid University, Abha 62529, Saudi Arabia; malmagbol@kku.edu.sa; 3 Dow International Dental College, Dow University of Health Science, Karachi 74200, Pakistan; eisha.abrar@duhs.edu.pk; 4 Orthodontic Department, Qurayyat Specialized Dental Center, Al-Qurayyat 77453, Saudi Arabia; moh.alruwaily@gmail.com (M.R.A.); m.jobab@gmail.com (M.A.A.)

**Keywords:** plaque scores, orthodontics, inflammatory cytokines, plant-based mouthwash

## Abstract

*Background and Objectives*: To investigate the antiplaque properties of two plant-based mouthwashes, *Morinda citrifolia* (MC) and *Ocimum sanctum* (OS), and their effect on TNF-α, IL-α, IL-β, IL-2, and IL-6 in gingival crevicular fluid (GCF) of patients undergoing fixed orthodontic treatment. *Materials and Methods*: Seventy-five individuals were recruited according to defined inclusion and exclusion criteria. This study was structured into two distinct phases. Phase I was a combination of toothbrushing using toothpaste containing fluoride (Protocol A), while Phase II toothbrushing included fluoride toothpaste and use of a mouthwash (Protocol B). For Phase II, individuals participating in this study were allocated into different groups through a randomization process: Group 1—0.12% CHX, Group 2—5% MC, and Group 3—4% OS. Each individual’s Phase I and Phase II scores were assessed. GCF was measured in three phases to determine the level of inflammatory biomarkers. The paired *t*-test evaluated the disparities between the pre- and post-plaque index. Categorical data were subjected to crosstab analysis to assess qualitative variables. The mean values of cytokine levels were presented. An unpaired *t*-test was employed to assess the levels of cytokines between individuals in Phase I and Phase II. *Results*: Toothbrushing, fluoride toothpaste, and the supplementary use of mouthwash (Phase II) resulted in mean plaque scores significantly lower than group A (*p* < 0.001). Cytokines TNF-α, IL-α, and IL-β demonstrated a significant downward trend in herbal mouthwash users. *Conclusions*: In conjunction with fluoridated toothpaste and brushing, OS and MC can serve as a viable alternative to conventional synthetic mouthwash CHX. This combination demonstrates reducing mean plaque scores and diminishing the levels of cytokines TNF-α, IL-α, and IL-β.

## 1. Introduction

Currently, malocclusion holds the position of third most prevalent oral health issue [1]. In the past few decades, there has been a noticeable rise in the need for orthodontic treatment among both children and adults. This increase can be linked to factors such as a greater awareness of dental aesthetics and oral health, advancements in orthodontic methods, and a cultural shift towards emphasizing personal appearance and self-improvement [2,3]. While orthodontic techniques aim to be minimally intrusive, the placement of orthodontic appliances on teeth frequently results in increased bacterial colonization in both the apparent and concealed biofilm on tooth surfaces [4,5]. As a result, the accumulation of bacteria has a role in the inflammation of the gum tissue that occurs during orthodontic treatment [6]. The relevance of dental biofilm buildup on fixed orthodontic appliances is underscored by the detection of an aerobic and anaerobic bacterial presence in blood samples collected from persons wearing such products [7]. This discovery underscores the necessity of integrating mechanical toothbrushing with chemical agents to achieve optimal plaque reduction in similar situations [8].

A range of chemical compounds with antiseptic or antibacterial qualities is employed to impede the development of plaque on the gum line, leading to the onset of gingivitis [9,10]. Chlorhexidine (CHX) is widely recognized as the most efficacious antibacterial mouthwash for inhibiting plaque formation and reducing the risk of gingivitis [11]. For more than thirty years, this particular cationic bis-biguanide has been widely regarded as a highly preferred broad-spectrum antiseptic [12]. Nevertheless, the frequent utilization of mouthwashes containing chlorhexidine (CHX) has been linked to many adverse effects, such as xerostomia (dryness of the mouth), a sense of burning, alterations in taste perception, dental discoloration (manifesting as brown staining), and detachment of the oral mucosa [13].

Within the domain of Ayurvedic medicine, there has been a recent focus on exploring the possible advantages of *Ocimum sanctum* (OS), also referred to as Holy Basil or Tulsi, for enhancing dental health [14]. Ongoing research in this field is actively investigating the potential of OS to mitigate plaque formation and improve oral hygiene, as suggested by certain studies [15,16]. Multiple research investigations have produced favorable outcomes concerning the influence of oral hygiene practices on oral health [17,18]. Nevertheless, the efficacy of this intervention in the specific population of orthodontic patients has yet to be thoroughly investigated and documented. In a similar vein, *Morinda citrifolia* (MC), also known as noni, exhibits a wide array of beneficial characteristics, including antifungal, antibacterial, analgesic, anti-inflammatory, antidiabetic, immunomodulatory, and antioxidative qualities [19]. Noni, a unique dietary ingredient, has been officially acknowledged by the European Commission. Since 2003, the marketing of noni, notably in the form of a wellness beverage, has been sanctioned [20]. The study conducted by Glang et al. examined the effects of MC on periodontal health and found a significant improvement in periodontal outcomes associated with its usage [19]. In light of the favorable results documented in these investigations, it is crucial to emphasize the necessity of additional research to carry out a thorough evaluation of the potential therapeutic uses of phytotherapeutic substances like noni, particularly among individuals receiving orthodontic treatment.

Presently, commercially accessible synthetic medicated mouthwashes demonstrate efficacy; yet their utilization is impeded by adverse effects and financial burdens. Evidence is scarce, comparing different synthetic mouthwashes and herbal alternatives in terms of their efficacy in reducing plaque accumulation among orthodontic patients and their impact on inflammatory biomarkers. Hence, the primary objective of this research was to assess and juxtapose the antiplaque properties of a common synthetic mouthwash (CHX) with two herbal-based mouthwashes (MC and OS) and to distinguish the adverse effects derived from herbal and synthetic sources in patients undergoing orthodontic treatment. The assessment also encompassed an examination of the effects of these oral rinses on the level of inflammatory cytokines. The hypothesis suggests that employing CHX mouthwash would result in enhanced antiplaque effectiveness, a reduction in inflammatory cytokine levels, and minimal adverse effects.

## 2. Material and Methods

The current investigation involved a randomized, controlled trial designed to explore the impacts of fixed orthodontic appliances. A cohort of 75 individuals undergoing orthodontic treatment at Health Sciences University (HSU) were thoughtfully recruited for this study. This study’s research protocols, materials, and methodologies underwent scrutiny and received approval from the Ethics Research Committee (ERC) with the IRB#3625-147. This study was carried out in full accordance with the ethical guidelines set forth by the World Medical Association for Human Experimentation, as outlined in the 2008 edition of the Helsinki Declaration [21]. Furthermore, this study’s procedures were conducted following the guidelines set forth by the Consolidated Standards of Reporting Trials (CONSORT), thereby ensuring a comprehensive and standardized approach to reporting the outcomes of the clinical trial [22].

Informed permission was obtained from all subjects participating in this study. This study’s inclusion criteria consisted of persons aged from 13 to 37 years who demonstrated sufficient oral hygiene, as determined by a plaque index of 20% or less. This assessment was conducted using the O’Leary index with the aid of a plaque-disclosing agent. Individuals who had utilized any oral rinse or consumed antibiotics in the week leading up to the initial assessment were not included in the research. The exclusion criteria included those who exhibited mouth breathing, were pregnant, smoked, had allergies or intolerance to herbal or synthetic mouthwashes, or had any systemic illness that could potentially impact their dental health. The trial’s cessation criteria encompassed severe overall gingival inflammation, the presence of significant underlying medical conditions, the utilization of antibiotics throughout the duration of this study, and instances in which a participant deviated from the prescribed protocol for more than three consecutive days [23].

The study was structured into two distinct phases. Phase I incorporated a combination of toothbrushing and the application of toothpaste containing fluoride (Protocol A), while Phase II extended this regimen to encompass toothbrushing, fluoride toothpaste, and the supplementary use of mouthwash (Protocol B). An evaluation was conducted that compared the Phase I and Phase II scores for each individual, accounting for the inherent diversity in oral hygiene practices across participants. This approach was designed to mitigate any potential influence arising from the Hawthorne effect and to accommodate variations in individual responses among subjects. Each phase of the observation period was assigned three weeks. The selection of this specific timeframe was based on the need for a period from 9 to 21 days to achieve an appropriate and measurable buildup of plaque within the oral cavity [23].

**Figure 1:**
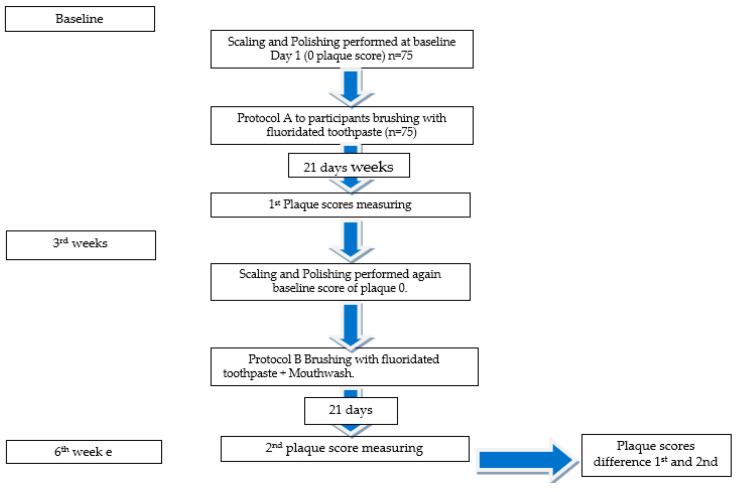
Flow diagram determining the methodology.

**Figure 2:**
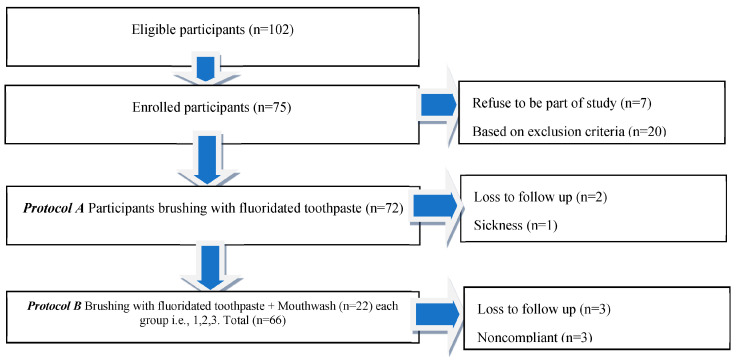
Flow diagram following CONSORT guidelines.

On day 21 of Phase I, all participants underwent an assessment for primary plaque scoring, which was conducted by an examiner who was unaware of the individuals’ identities. The examiner employed a revealing agent and the bonded bracket plaque index for this evaluation. This study involved the analysis of scores acquired from a total of eight teeth, with two teeth being picked from each quadrant. Subsequently, the scores were documented in a specifically designated data sheet. The ultimate score was determined by computing the average of the scores obtained from all eight teeth. Following the initial intervention grading, the participants were then referred for scaling and polishing procedures to obtain a baseline plaque score of zero. During standard orthodontic treatment, the elastomeric O-rings were substituted. Following the established protocols, the individuals under study were allocated into different groups via a randomization process. Group 1—0.12% CHX, Group 2—5% MC, and Group 3—4% OS (Figure 1 and Figure 2).

## 3. Preparation of 5% MC Mouthwash

The process of creating a mouthwash with a 5% concentration of MC involved several sequential phases. The fruit underwent an initial process of cleansing and desiccation at a temperature of 400 °C. After undergoing the drying process, the substance was mixed with ethanol of a 96% concentration. To obtain a more concentrated consistency, a rotary evaporator was utilized. A total of ten grams of MC extract was subsequently employed in the formulation of the mouthwash, resulting in a concentration of Noni at 5%. Various components were incorporated into the Noni extract, including 2 mg of blue dye, 200 mg of sodium benzoate, 100 mg of acidum benzoate, 200 mg of Tween80, 600 mg of sodium saccharin, 2 mL of oleum methane, and an appropriate amount of aqua dest to achieve a final volume of 200 mL. The formulation of the MC mouthwash with a 5% concentration was a direct outcome of this meticulous composition. Consistent with the aforementioned results, the present investigation utilized a comparable concentration of 5% Noni mouthwash to evaluate its efficacy [19,24].

## 4. Preparation of 4% OS Mouthwash

The procedure was initiated by meticulously pulverizing desiccated leaves of *Ocimum sanctum* into a powdered form. Subsequently, a quantity of 300 g of finely powdered *O. sanctum* material was subjected to maceration with 100% ethanol in a round-bottom flask for one week. The extract produced during the maceration phase underwent a filtration procedure. The extract was initially strained using a muslin cloth to eliminate any large particles, followed by filtration through Whatman No. 1 filter paper to improve its transparency. Subsequently, the filtrate that was acquired underwent a meticulous reduction process at a temperature lower than 50 °C, leading to the formation of a solid residue comprising the concentrated extract of *O. sanctum*. The experiment commenced with the utilization of 300 g of *O. sanctum* powder, which was dissolved in 1 L of ethanol. Subsequently, the procedure resulted in the production of 18 g of residue, also referred to as the extract. This outcome corresponds to a yield of 6% *w*/*w* (weight-to-weight). To attain a final concentration of 4% (*w*/*v*), the extract was blended with a mixture of polyethylene glycol 400 (20% *v*/*v*) and sterile distilled water [25,26].

To maintain consistency among all groups of mouthwash, the definitive extracts from both variants of herbal mouthwash were forwarded to a pharmaceutical company. A set of improvements was implemented within the organizational structure of the corporation. The alterations involved the integration of a synthetic dye (blue), the introduction of antibacterial substances (sodium benzoate), and the inclusion of a taste enhancer (mint). The selection of mint as an ingredient was motivated by its inclusion in synthetic mouthwash formulations, which allowed a uniform flavor characteristic to be maintained. The aforementioned modifications were implemented with the explicit intention of obscuring the inherent hue and characteristics of the extracts obtained. The incorporation of the specified constituents led to the maintenance of consistency in the ultimate compositions of mouthwash, while simultaneously integrating the intended characteristics.

## 5. Mouthwash Rinse Instructions to Participants

The individuals in each experimental group were instructed to utilize a volume of 10 mL of the mouthwash offered, which was thereafter diluted with an equivalent quantity of water as per the manufacturer’s guidelines. The suggested time frame for carrying out this rinse protocol was 60 s, to be executed on two occasions during 24 h. The first occurrence was scheduled to occur after their morning meal, while the second was planned to take place before bedtime. It is recommended to observe a minimum waiting period of one hour after completing oral hygiene practices such as toothbrushing and toothpaste application before utilizing mouthwash. Furthermore, in light of the potential interaction between chlorhexidine and sodium lauryl sulfate (SLS), a commonly used detergent in toothpaste, as well as fluoride, precautionary measures were taken to prohibit the use of the mouthwash within the period of 0.5 to 2 h after applying toothpaste [23].

## 6. Evaluation of Different Mouthwash Experiences by Participants and Assessment of Plaque Scores

Participants were regularly reminded of the study protocols through weekly phone calls to ensure their consistent adherence. Additionally, participants were recalled after three weeks to provide feedback on their experiences with the mouthwashes, addressing aspects such as taste alteration, dryness, and any potential side effects.

## 7. GCF Collection, Measuring, and Inflammatory Cytokine Analysis

GCF was measured in three phases: baseline, Phase I, and Phase II. The selection of the sample collection site was intentionally targeted towards a region characterized by less gingival inflammation. The constant placement identified across all participants was close to the canines within the upper dental arch. To establish consistent conditions, the gathered samples underwent the process of homogenization. Sterilized gauze was utilized as a means of achieving effective isolation, hence mitigating the risk of contamination. To obtain gingival crevicular fluid (GCF), a pipette was carefully placed at the gingival sulcus, ensuring a delicate connection was established at the gingival border. The objective was to utilize a non-intrusive methodology for the acquisition of genomic cell-free DNA (GCF) while minimizing interference, with a specific focus on obtaining a collection volume of 1 µL. Any pipette that came into contact with blood or saliva throughout the operation was eliminated from the analysis. GCF was thereafter transferred, with caution, into Eppendorf tubes with a volume of 0.5 mL. Subsequently, centrifugation was conducted at a velocity of 3000 revolutions per minute (rpm), and the samples were held at a temperature of −80 °C for 10 min, until the commencement of the assay. Throughout these operations, rigorous blinding protocols were implemented to guarantee impartial and unbiased results. The quantification of cytokines was conducted by a biologist within the biochemistry laboratory. Significantly, the biologist maintained a state of blindness regarding the experimental conditions, ensuring an impartial approach. The level of concordance, represented by a kappa coefficient of 0.77, was ascertained. The analysis was centered on examining specific inflammatory biomarkers, namely TNF-α, IL-1α, IL-2,6, and IL-β. The quantification of these biomarkers was performed using the enzyme-linked immunosorbent assay (ELISA) method, following the guidelines provided by the manufacturer. The assessment of cytokines in the collected samples was undertaken consistently and reliably by following these recommendations. ELISA demonstrated a high sensitivity of over 99.1% for all cytokines present in the GCF [27].

### Statistical Analysis

The data analysis was conducted using SPSS IBM v21 software (IBM; Armonk, NY, USA). The paired *t*-test was utilized to evaluate the disparities between the pre-plaque index and post-plaque index across all groups. To facilitate a more extensive examination of the impacts of the interventions, we evaluated the mean disparity in the post-plaque index by conducting comparisons both between and within groups utilizing the post hoc Tukey test. Categorical data were subjected to crosstab analysis to assess qualitative variables. The mean values of cytokine levels were presented. An unpaired *t*-test was employed to assess the levels of cytokines between individuals of Phases I and II.

## 8. Results

The number of participants enrolled in this study was 102. The number of participants eligible for the study was 75 (Figure 2). Data related to the age of subjects and first and second plaque scores were found to be distributed normally. The number of subjects who completed this study was 66. The number of females (*n* = 48) was higher compared to men (*n* = 18). The majority of participants finished basic school (*n* = 47) (Table 1).

Following the combination of toothbrushing and the application of toothpaste containing fluoride (Phase I), participants displayed comparable plaque scores (*p* > 0.001). Similarly, toothbrushing, fluoride toothpaste, and the supplementary use of mouthwash (Phase II) resulted in mean plaque scores significantly lower than those of group A (*p* < 0.001). The mean plaque scores of participants with CHX (19.26 ± 2.11) were significantly higher than MC (15.71 ± 2.27) and OS (16.33 ± 1.98) mouthwashes after protocol B (*p* < 0.001). Both herbal mouthwash MC (15.71 ± 2.27) and OS (16.33 ± 1.98) demonstrated comparable mean plaque scores (*p* > 0.001) (Table 2).

Table 3 demonstrates the subjective experience of participants after the use of different mouthwashes. Mouth burning, mouth dryness, unpleasant taste, and unfreshness were highly reported in participants who used 0.12% CHX. However, participants who used MC and OS as mouthwash were more content in terms of mouth burning, mouth dryness, unpleasant taste, and freshness.

Table 4 exhibits levels of inflammatory cytokines in GCF at baseline, Phase I, and Phase II of the trial. Participants exposed to Protocol A toothbrushing and the application of toothpaste containing fluoride demonstrated a decrease in inflammatory cytokines from baseline. Similarly, when participants were exposed to protocol B toothbrushing, fluoride toothpaste, and the supplementary use of mouthwash, a downward trend was observed in all inflammatory cytokines. However, cytokines TNF-α, IL-α, and IL-β demonstrated a significant downward trend in herbal mouthwash users compared to CHX mouthwash users (Table 4).

## 9. Discussion

In this study, the hypothesis centered on the notion that utilizing CHX mouthwash would lead to improved antiplaque effectiveness, decreased inflammatory cytokine levels, and limited adverse effects. However, this hypothesis was entirely refuted because the application of herbal mouthwashes (OS and MC) showcased superior antiplaque efficacy. Furthermore, these herbal alternatives demonstrated a noteworthy reduction in inflammatory cytokines—TNF-α, IL-α, and IL-β—accompanied by a more favorable subjective experience, including taste, mouth dryness, mouth burning, and a sense of freshness. This was in contrast to the outcomes observed with CHX.

A recent study indicated that herbal mouthwash exhibited notably lower plaque scores compared to synthetic mouthwash [23,28]. The average plaque scores observed for both types of herbal mouthwash were similar. The observed reduction in plaque scores among participants who used the herbal mouthwash can be attributed to the potent antibacterial and anti-inflammatory properties present in OS and MC. OS contains a range of phytochemicals, including flavonoids, polyphenols, and essential oils. These bioactive compounds contribute to its dual characteristics of being both an antioxidant and an anti-inflammatory agent, properties which collectively work to alleviate oxidative stress [15,25]. Furthermore, research indicates that OS can hinder the adhesion of bacteria to dental surfaces, thereby intervening at the initial stages of plaque development [17]. Similarly, MC, also known as Noni, is endowed with various bioactive compounds known for their health benefits. These compounds include flavonoids like quercetin and kaempferol, along with coumarins like scopoletin. These components are recognized for their antioxidative and anti-inflammatory properties, which are significant for managing overall health, including oral health [24,29]. An essential aspect of Noni lies in its iridoids, a distinctive class of compounds. Iridoids have garnered attention due to their multifaceted effects, including antinociceptive (pain-relieving) and anti-inflammatory actions. Additionally, iridoids exhibit potent antibacterial properties, which contribute to inhibiting bacterial growth and thus have the potential to impact disease progression [19,24]. Overall, the findings of this study underscore the effectiveness of herbal mouthwashes in reducing plaque scores, primarily due to their antibacterial and anti-inflammatory characteristics. The synergistic contributions of the phytochemicals in both OS and MC play pivotal roles in curbing plaque buildup [18,30]. In contrast, a study conducted by Singh et al. revealed that chlorhexidine (CHX) has shown superior efficacy in inhibiting the regrowth of plaque when compared to herbal mouthwashes [31]. Multiple factors may contribute to the variances in outcomes observed, including potential differences among individuals. These differences may involve various elements such as cultural influences, eating habits, and regional peculiarities. An additional factor that may account for these contradictory results could be ascribed to disparities in the methodology, including changes in brushing techniques, timing of toothbrushing, and the specific toothpaste employed [32].

The levels of the inflammatory biomarkers TNF-α, IL-α, and IL-β exhibited a significant reduction in participants who used the herbal mouthwash as compared to those who did not. This reduction was particularly noteworthy in the group that used the herbal mouthwash containing OS and MC. OS and MC have been subject to investigation due to their potential to decrease the production of TNF-α, a prominent cytokine associated with inflammation [33,34]. TNF-α plays a pivotal role in promoting inflammatory processes and immune responses [35]. The potential of Holy Basil to restrain or lower the production of TNF-α holds promise in mitigating inflammatory reactions within the body. Furthermore, interleukins IL-α and IL-β are also recognized as proinflammatory cytokines and are actively involved in regulating immune responses and inflammation [36]. Research has indicated that the bioactive constituents within OS and MC, such as flavonoids and polyphenols, can modulate the production of IL-α and IL-β [37]. These modulatory effects have the potential to attenuate the intensity of the inflammatory response [38,39]. Other inflammatory cytokines IL-2 and IL-6 exhibited comparable levels of cytokines with synthetic CHX mouthwash.

The higher occurrence of taste alteration observed with chlorhexidine (CHX) usage is in line with findings from studies conducted by Gürgan, Marinone, and Savoldi [40,41]. Both of these studies concluded that concentrations of CHX exceeding 0.12% are linked to taste alteration. Similarly, our investigation into the sensations of burning and dryness in the mouth aligns with results from a study carried out by Asiri et al. [42]. In this study, we noted that more than 80% of participants in the CHX group reported experiencing a burning sensation. This correspondence in outcomes highlights the potential discomfort associated with CHX use, especially when compared to the alternative herbal options. The study conducted was indeed an example of a randomized controlled trial (RCT), which holds significant value due to its ability to establish causal relationships, minimize bias, generalize findings to wider populations, and provide strong evidence to inform decision-making in various fields, particularly in healthcare and social sciences. Nevertheless, it is imperative to acknowledge that, its commendable attributes notwithstanding, the present investigation possesses certain constraints. Additional research is necessary to evaluate several characteristics, such as substantivity, safety, plaque reduction, and antibacterial activity, to enhance our findings. Before contemplating the incorporation of these herbal mouthwashes with other agents for routine plaque removal techniques, it is crucial to conduct further inquiry.

## Figures and Tables

**Table 1:** Demographics of participants.

Characteristics	Interventional Group			
Gender	**Group 1: 0.2% CHX**	**Group 2: 5% MC**	**Group 3: 4% OS**	**Total**
Male	6	8	4	18
Female	16	14	18	48
Age (mean ± SD)	19.25 ± 1.58	20.66 ± 1.29	19.98 ± 1.36	
Education				
Basic school	14	18	15	47
High school	6	2	4	12
Bachelor’s	1	1	2	4
Masters	1	1	1	3

**Table 2:** Plaque scores and comparison between and within interventional groups.

Plaque Scores (Mean ± SD)	Group 1: 0.2% CHX	Group 2: 5% MC	Group 3: 4% OS	*p*-Value
First score (Protocol A)	33.25 ± 4.26	32.29 ± 3.97	31.59 ± 4.01	
Second Score (Protocol B)	19.26 ± 2.11	15.71 ± 2.27	16.33 ± 1.98	
Tukey Post Hoc				
Group 1: 0.12% CHX	-	-	-	0.007
Group 2: 5% MC	0.011 *	-	-	
Group 3: 4% OS	0.013 *	0.289	-	

* Significant diffrence.

**Table 3:** The subjective experience of participants after using different mouthwashes.

Symptoms	Intervention Groups			
	Group 1: 0.2% CHX	Group 2: 5% MC	Group 3: 4% OS	Total
Taste Changes				
Yes	18	2	1	21
No	4	20	21	45
Mouth Burning				
Yes	16	-	4	20
No	6	22	18	46
Mouth Dryness				
Yes	14	1	5	20
No	8	21	17	46
Taste				
Pleasant	2	15	16	33
Unpleasant	20	7	6	33
Feeling of Freshness				
Yes	10	17	18	45
No	12	5	4	21

**Table 4:** Levels of inflammatory cytokines in GCF at baseline, Phase I, and Phase II of the trial.

Inflammatory Biomarkers in GCF	Mean ± SD Baseline	Mean ± SD 3 Weeks (Protocol A) Brushing and Fluoridated Toothpaste	Groups	Mean ± SD 6 Weeks (Protocol B) Brushing and Fluoridated Toothpaste	*p*-Value
TNF-α (pg/mL)	6.32 ± 0.65	5.27 ± 0.27	0.12% CHX	4.25 ± 0.63	0.030
			5% MC	2.99 ± 0.75	0.010 *
			4% OS	2.79 ± 1.01	0.020 *
IL-α (pg/mL)	0.084 ± 0.06	0.077 ± 0.03	0.12% CHX	0.058 ± 0.002	0.270
			5% MC	0.021 ± 0.001	0.020 *
			4% OS	0.020 ± 0.005	0.030 *
IL-β (pg/mL)	8.69 ± 0.25	7.14 ± 0.33	0.12% CHX	7.02 ± 0.35	0.450
			5% MC	6.15 ± 0.15	0.040 *
			4% OS	6.10 ± 0.22	0.010 *
IL-2 (pg/mL)	3.5 ± 1.21	3.0 ± 1.01	0.12% CHX	2.79 ± 0.87	0.660
			5% MC	2.23 ± 0.65	0.740
			4% OS	2.10 ± 0.89	0.890
IL-6 (pg/mL)	3.85 ± 1.01	3.26 ± 1.30	0.12% CHX	2.95 ± 1.36	0.550
			5% MC	2.98 ± 0.98	0.980
			4% OS	2.85 ± 0.33	0.780

* unpaired *t*-test shows statistically significant.

## Data Availability

Can be made available on request.

## References

[1] Gudipaneni R.K., Aldahmeshi R.F., Patil S.R., Alam M.K. (2018). The Prevalence of Malocclusion and the Need for Orthodontic Treatment among Adolescents in the Northern Border Region of Saudi Arabia: An Epidemiological Study. BMC Oral Health.

[2] Meyer-Marcotty P., Klenke D., Knocks L., Santander P., Hrasky V., Quast A. (2021). The Adult Orthodontic Patient over 40 Years of Age: Association between Periodontal Bone Loss, Incisor Irregularity, and Increased Orthodontic Treatment Need. Clin. Oral Investig..

[3] Grewal H., Sapawat P., Modi P., Aggarwal S. (2019). Psychological Impact of Orthodontic Treatment on Quality of Life—A Longitudinal Study. Int. Orthod..

[4] Sharma R., Sharma K., Sawhney R. (2018). Evidence of Variable Bacterial Colonization on Coloured Elastomeric Ligatures during Orthodontic Treatment: An Intermodular Comparative Study. J. Clin. Exp. Dent..

[5] Akgun O.M., Altug H., Karacay S., Guven Polat G., Duyan S., Bedir O. (2014). Effect of 2 Elastomeric Ligatures on Microbial Flora and Periodontal Status in Orthodontic Patients. Am. J. Orthod. Dentofac. Orthop..

[6] Condò R., Casaglia A., Condò S.G., Cerroni L. (2012). Plaque Retention on Elastomeric Ligatures. An in Vivo Study. Oral Implantol..

[7] Aghili H., Jafari Nadoushan A.A., Herandi V. (2015). Antimicrobial Effect of Zataria Multiflora Extract in Comparison with Chlorhexidine Mouthwash on Experimentally Contaminated Orthodontic Elastomeric Ligatures. J. Dent..

[8] Oshagh M., Dashliborun Y.N., Saravi M.E., Bazargani A. (2014). Evaluation of Chlorhexidine and Zatariamultiflora Essential Oil in Removing Streptococcus Viridans and Candida from the Surface of Removable Orthodontic Appliances: A Randomized Clinical Trial. J. Maz. Univ. Med. Sci..

[9] Kharaeva Z.F., Mustafaev M.S., Khazhmetov A.V., Gazaev I.H., Blieva L.Z., Steiner L., Mayer W., de Luca C., Korkina L.G. (2020). Anti-Bacterial and Anti-Inflammatory Effects of Toothpaste with Swiss Medicinal Herbs towards Patients Suffering from Gingivitis and Initial Stage of Periodontitis: From Clinical Efficacy to Mechanisms. Dent. J..

[10] Szliszka E., Czuba Z.P., Domino M., Mazur B., Zydowicz G., Krol W. (2009). Ethanolic Extract of Propolis (EEP) Enhances the Apoptosis- Inducing Potential of TRAIL in Cancer Cells. Molecules.

[11] Feres M., Gursky L.C., Faveri M., Tsuzuki C.O., Figueiredo L.C. (2009). Clinical and Microbiological Benefits of Strict Supragingival Plaque Control as Part of the Active Phase of Periodontal Therapy. J. Clin. Periodontol..

[12] Haydari M., Bardakci A.G., Koldsland O.C., Aass A.M., Sandvik L., Preus H.R. (2017). Comparing the Effect of 0.06–0.12% and 0.2% Chlorhexidine on Plaque, Bleeding and Side Effects in an Experimental Gingivitis Model: A Parallel Group, Double Masked Randomized Clinical Trial. BMC Oral Health.

[13] Ajay Rao H., Bhat S., Hegde S., Jhamb V. (2014). Efficacy of Garlic Extract and Chlorhexidine Mouthwash in Reduction of Oral Salivary Microorganisms, an in Vitro Study. Anc. Sci. Life.

[14] Cohen M.M. (2014). Tulsi—*Ocimum sanctum*: A Herb for All Reasons. J. Ayurveda Integr. Med..

[15] Gupta D., Bhaskar D.J., Gupta R.K., Karim B., Jain A., Singh R., Karim W. (2014). A Randomized Controlled Clinical Trial of *Ocimum sanctum* and Chlorhexidine Mouthwash on Dental Plaque and Gingival Inflammation. J. Ayurveda Integr. Med..

[16] Hosamane M., Acharya A.B., Vij C., Trivedi D., Setty S.B., Thakur S.L. (2014). Evaluation of Holy Basil Mouthwash as an Adjunctive Plaque Control Agent in a Four Day Plaque Regrowth Model. J. Clin. Exp. Dent..

[17] Penmetsa G., Pitta S. (2019). Efficacy of *Ocimum sanctum*, Aloe Vera and Chlorhexidine Mouthwash on Gingivitis: A Randomized Controlled Comparative Clinical Study. Ayu.

[18] Deepika B.A., Ramamurthy J., Girija S., Jayakumar N.D. (2022). Evaluation of the Antimicrobial Effect of *Ocimum sanctum* L. Oral Gel against Anaerobic Oral Microbes: An In Vitro Study. World J. Dent..

[19] Glang J., Falk W., Westendorf J. (2013). Effect of *Morinda citrifolia* L. Fruit Juice on Gingivitis/Periodontitis. Mod. Res. Inflamm..

[20] Semeniuc C.A., Rotar M.A., Suharoschi R., Tofana M., Muste S. (2011). Overview of the EU Legislation on Novel Foods and Novel Food Ingredients. Agriculture.

[21] Goodyear M.D.E., Krleza-Jeric K., Lemmens T. (2007). The Declaration of Helsinki. Br. Med. J..

[22] Schulz K.F., Altman D.G., Moher D. (2010). CONSORT 2010 Statement: Updated Guidelines for Reporting Parallel Group Randomised Trials. BMC Med..

[23] Niazi F.H., Kamran M.A., Naseem M., AlShahrani I., Fraz T.R., Hosein M. (2018). Anti-Plaque Efficacy of Herbal Mouthwashes Compared to Synthetic Mouthwashes in Patients Undergoing Orthodontic Treatment: A Randomised Controlled Trial. Oral Health Prev. Dent..

[24] Huang H.L., Liu C.T., Chou M.C., Ko C.H., Wang C.K. (2015). Noni (*Morinda citrifolia* L.) Fruit Extracts Improve Colon Microflora and Exert Anti-Inflammatory Activities in Caco-2 Cells. J. Med. Food.

[25] Bhat S.S., Kochikar Pai R., Salman A., Chandra J. (2015). Use of an Extract of Indian Sacred Plant *Ocimum sanctum* as an Anticariogenic Agent: An in Vitro Study. Int. J. Clin. Pediatr. Dent..

[26] Agarwal P., Nagesh L. (2010). Murlikrishnan Evaluation of the Antimicrobial Activity of Various Concentrations of Tulsi (*Ocimum sanctum*) Extract against Streptococcus Mutans: An in Vitro Study. Indian J. Dent. Res..

[27] Kamran M.A., Alnazeh A.A., Almagbol M., Almoammar S., Alhaizaey A.H.A., Alshahrani I. (2023). Role of Six Cytokines and Bone Metabolism Biomarkers in Gingival Crevicular Fluid in Patients Undergoing Fixed Orthodontic Appliance Treatment in Comparison with Aligners: A Clinical Study. Angle Orthod..

[28] Sharma R., Hebbal M., Ankola A., Murugaboopathy V., Shetty S. (2014). Effect of Two Herbal Mouthwashes on Gingival Health of School Children. J. Tradit. Complement. Med..

[29] Talattof Z., Azad A., Zahed M., Shahradnia N. (2018). Antifungal Activity of Xylitol against Candida Albicans: An in Vitro Study. J. Contemp. Dent. Pract..

[30] Zaini W.S. (2021). Antibacterial Effectiveness of *Morinda citrifolia* L. Extract on Salmonella Typhi Bacteria Using Serial Dilution Method with 15–60 Minutes Contact Time. Pharmacogn. J..

[31] Singh A., Daing A., Dixit J. (2013). The Effect of Herbal, Essential Oil and Chlorhexidine Mouthrinse on de Novo Plaque Formation. Int. J. Dent. Hyg..

[32] Quintas V., Prada-López I., Donos N., Suárez-Quintanilla D., Tomás I. (2015). Antiplaque Effect of Essential Oils and 0.2% Chlorhexidine on an in Situ Model of Oral Biofilm Growth: A Randomised Clinical Trial. PLoS ONE.

[33] Ren Y., Hazemeijer H., de Haan B., Qu N., de Vos P. (2007). Cytokine Profiles in Crevicular Fluid During Orthodontic Tooth Movement of Short and Long Durations. J. Periodontol..

[34] Tatullo M., Codispoti B., Paduano F., Nuzzolese M., Makeeva I. (2019). Strategic Tools in Regenerative and Translational Dentistry. Int. J. Mol. Sci..

[35] Bonato R.C.S., Mapengo M.A.A., De Azevedo-Silva L.J., Janson G., De Carvalho Sales-Peres S.H. (2022). Tooth Movement, Orofacial Pain, and Leptin, Interleukin-1β, and Tumor Necrosis Factor-α Levels in Obese Adolescents. Angle Orthod..

[36] Graves D.T., Cochran D. (2003). The Contribution of Interleukin-1 and Tumor Necrosis Factor to Periodontal Tissue Destruction. J. Periodontol..

[37] Mohammed A., Saidath K., Mohindroo A., Shetty A., Shetty V., Rao S., Shetty P. (2019). Assessment and Measurement of Interleukin 6 in Periodontal Ligament Tissues during Orthodontic Tooth Movement. World J. Dent..

[38] Başaran G., Özer T., Kaya F.A., Hamamci O. (2006). Interleukins 2, 6, and 8 Levels in Human Gingival Sulcus during Orthodontic Treatment. Am. J. Orthod. Dentofac. Orthop..

[39] Inchingolo F., Tatullo M., Marrelli M., Inchingolo A.M., Tarullo A., Inchingolo A.D., Dipalma G., Brunetti S.P., Tarullo A., Cagiano R. (2011). Combined Occlusal and Pharmacological Therapy in the Treatment of Temporo-Mandibular Disorders. Eur. Rev. Med. Pharmacol. Sci..

[40] Gürgan C.A., Zaim E., Bakirsoy I., Soykan E. (2006). Short-Term Side Effects of 0.2% Alcohol-Free Chlorhexidine Mouthrinse Used as an Adjunct to Non-Surgical Periodontal Treatment: A Double-Blind Clinical Study. J. Periodontol..

[41] Marinone M.G., Savoldi E. (2000). Chlorhexidine and Taste. Influence of Mouthwashes Concentration and of Rinsing Time. Minerva Stomatol..

[42] Asiri F.Y.I., Alomri O.M.H., Alghmlas A.S., Gufran K., Sheehan S.A., Shah A.H. (2016). Evaluation of Efficacy of a Commercially Available Herbal Mouthwash on Dental Plaque and Gingivitis: A Double-Blinded, Parallel, Randomized, Controlled Trial. J. Int. Oral Health.

